# Fibrin glue as a local drug-delivery system for bacteriophage PA5

**DOI:** 10.1038/s41598-018-38318-4

**Published:** 2019-02-14

**Authors:** Evgenii Rubalskii, Stefan Ruemke, Christina Salmoukas, Andrey Aleshkin, Svetlana Bochkareva, Evgeny Modin, Bakr Mashaqi, Erin C. Boyle, Dietmar Boethig, Maxim Rubalsky, Eldar Zulkarneev, Christian Kuehn, Axel Haverich

**Affiliations:** 1 Department of Cardiothoracic, Transplantation and Vascular Surgery, Hannover Medical School, Hannover, Germany; 2 Lower Saxony Centre for Biomedical Engineering, Implant Research and Development, Hannover, Germany; 3 Gabrichevsky Research Institute for Epidemiology and Microbiology, Moscow, Russia; 4 CIC nanoGUNE, Donostia, San- Sebastian, Spain; 5 Astrakhan State Medical University, Astrakhan, Russia

## Abstract

Fibrin glue has been used clinically for decades in a wide variety of surgical specialties and is now being investigated as a medium for local, prolonged drug delivery. Effective local delivery of antibacterial substances is important perioperatively in patients with implanted medical devices or postoperatively for deep wounds. However, prolonged local application of antibiotics is often not possible or simply inadequate. Biofilm formation and antibiotic resistance are also major obstacles to antibacterial therapy. In this paper we test the biocompatibility of bacteriophages incorporated within fibrin glue, track the release of bacteriophages from fibrin scaffolds, and measure the antibacterial activity of released bacteriophages. Fibrin glue polymerized in the presence of the PA5 bacteriophage released high titers of bacteriophages during 11 days of incubation in liquid medium. Released PA5 bacteriophages were effective in killing *Pseudomonas aeruginosa* PA01. Overall, our results show that fibrin glue can be used for sustained delivery of bacteriophages and this strategy holds promise for many antibacterial applications.

## Introduction

Effective local delivery of antibacterial substances is important perioperatively in patients with implanted medical devices or postoperatively for deep wounds. However, prolonged local application of antibacterial drugs for such patients is often impossible or inadequate. Different drug carriers have been tested for local drug-delivery with a prolonged release of antibiotics. A number of materials such as polymethylmethacrylate, collagen, chitosan, and polyethylene glycol have been loaded with antibiotics and applied intraoperatively thereby conferring a prolonged local antimicrobial effect^1–4^. Such a local drug delivery approach is especially important for patients with implanted cardiovascular devices because of the high mortality and healthcare costs associated with infectious complications^5,6^.

Conventional antibacterial drugs are becoming less effective due to the development and increasing incidence of antibiotic resistance and tolerance. The increased prevalence of antibiotic resistance has renewed worldwide interest in the use of bacteriophages^7^ – viruses that specifically infect bacteria. Bacteriophages bind to specific receptors on the bacteria cell surface, inject and replicate their genetic material, and release progeny upon lysis of the host cell. Bacteriophages were discovered approximately 100 years ago and have been safely and effectively used for infection therapy since 1919, most frequently in Russia and Eastern Europe^8,9^. Modern phage therapies range from the use of off-the-shelf formulations to customizable, personalized magistral preparations^7,10^. Bacteriophages are good candidates for antibacterial therapy as they are non-toxic, highly specific, and leave the normal microbiota undisturbed^11^. Importantly, bacteriophages are self-amplifying antimicrobials in that they replicate themselves as long as there are host bacteria to infect. Interestingly, some phages are particularly effective at penetrating and disrupting biofilms which are highly resistant to host immune defenses and the penetration of antibiotics^12^.

Fibrin glue is a two-component hemostat, sealant, and tissue adhesive consisting of fibrinogen and thrombin. It has been used clinically for decades in a wide variety of surgical specialties and also has several newer uses in cell and drug delivery^13^. Several studies have found that the incorporation of antibiotics into fibrin glue facilitates effective site-directed, sustained-release drug-delivery^14–17^ including during cardiovascular surgery^18,19^. The aim of this study was to determine whether conventional fibrin glue could be used as a local drug-delivery system for bacteriophages. The biocompatibility of bacteriophages within fibrin glue, the release of bacteriophages from fibrin scaffolds over time, and the antibacterial activity of released bacteriophages were tested. If proven effective, bacteriophages embedded in fibrin glue could serve to treat or prevent infections in many surgical fields, especially those infections associated with antibiotic resistant bacteria or biofilms.

## Results

**Figure 1:**
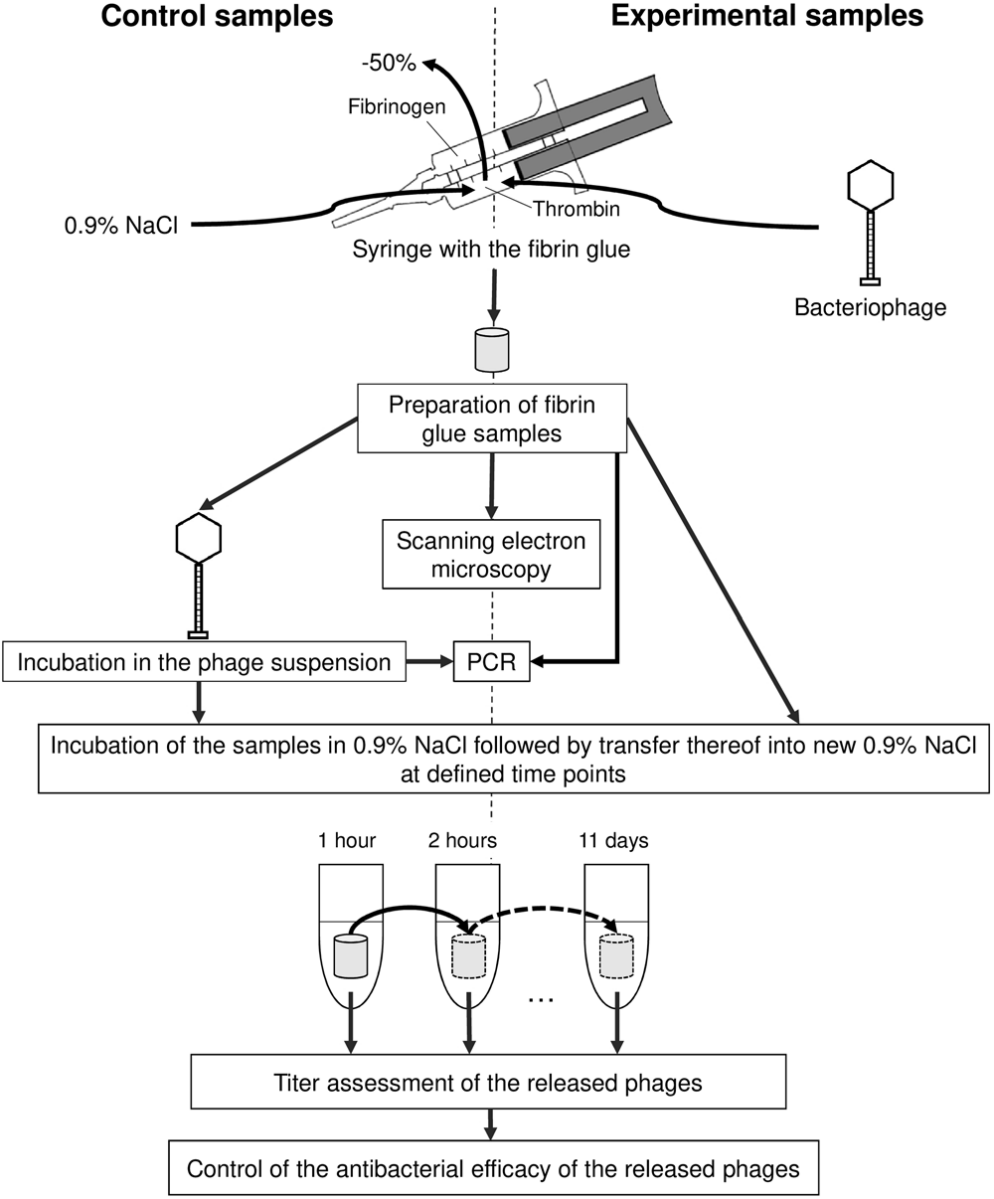
Schematic diagram of the experimental protocol.

**Figure 2:**
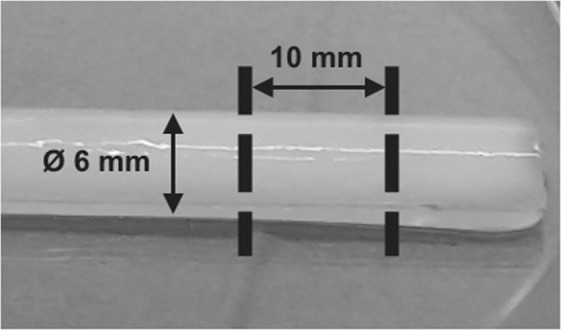
A polymerized fibrin glue scaffold prior to cutting into blocks.

**Figure 3:**
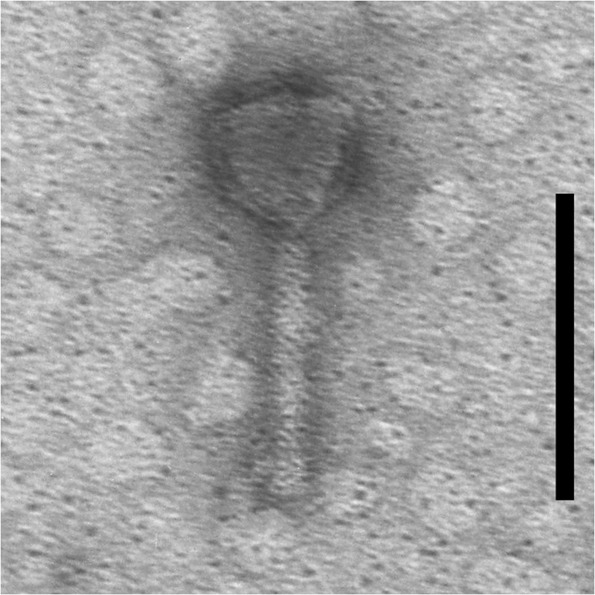
Transmission electron microscopy image of the PA5 phage. Scale bar, 100 nm.

The presented scheme (Fig. 1) outlines the procedures and experiments conducted to create fibrin glue scaffolds (Fig. 2) with and without the PA5 bacteriophage (Fig. 3) in order to investigate their properties.

### Incorporation and distribution of bacteriophage PA5 in fibrin glue scaffolds

**Figure 4:**
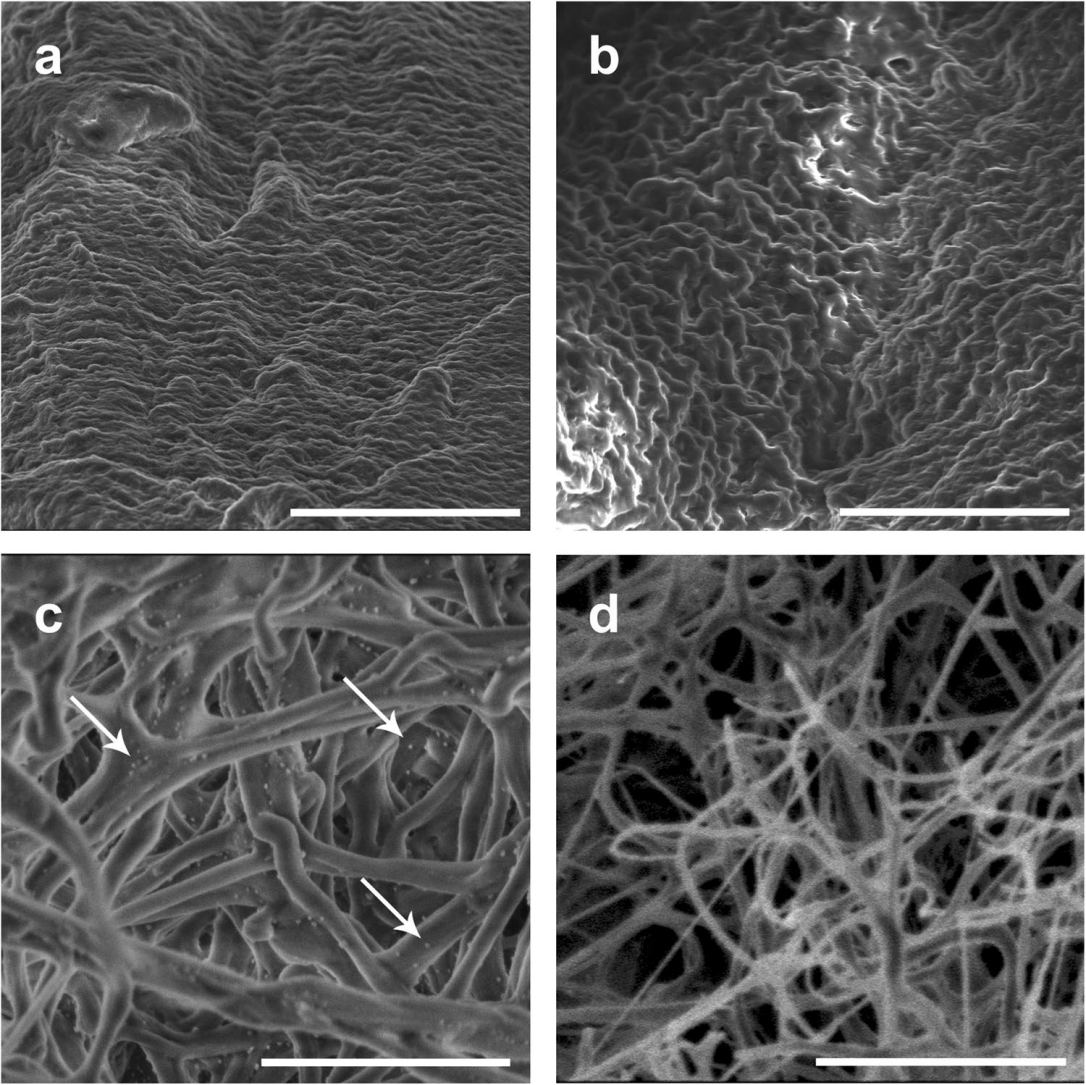
Electron microscopy of the outer surface of the fibrin glue blocks (**a** – experimental, **b** – control) and the inner architecture of the blocks (**c** – experimental, **d** – control). Scale bar a-b, 10 µm; scale bar c-d, 2 µm. Arrows indicate bacteriophage capsids associated with the fibrin fibers.

Fibrin glue blocks containing PA5 bacteriophages (experimental samples) or the normal saline solution alone (control samples) were examined by scanning electron microscopy (SEM) to investigate the incorporation and distribution of PA5 bacteriophages within fibrin glue scaffolds. The surface structure of the both experimental and control fibrin blocks did not show remarkable differences (Fig. 4a,b). Bacteriophage virions were not observed on the surface of the experimental blocks (Supplemental Fig. S1). The inner structure of both the experimental and control fibrin blocks showed well distinguishable fibrin fibers (Fig. 4c,d). Multiple bacteriophage capsids with a diameter of 40 ± 5 nm were clearly homogeneously distributed over the surface of the fibrin fibers (Fig. 4c).

### Release of bacteriophages from the fibrin glue

**Figure 5:**
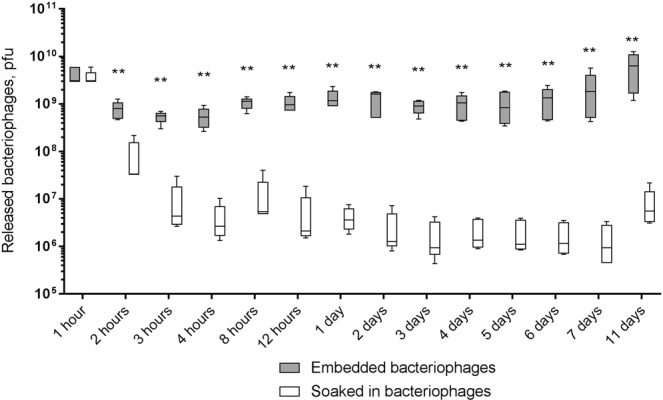
Measurement of *P*. *aeruginosa* PA5 phage release from fibrin glue blocks. Comparison of fibrin glue blocks with embedded bacteriophages to control samples where fibrin glue blocks were soaked in a solution of PA5 phage. The fibrin glue blocks were continuously incubated in the normal saline solution with permanent rotation. The solution was changed at each time point. Numbers of released phages are presented as the mean (n = 5) ± the standard deviation. *p < 0.05; **p < 0.01; ***p < 0.001.

Continuous incubation of the fibrin glue blocks in 0.9% NaCl was performed in order to estimate sustained phage release from fibrin scaffolds. Already one hour after submersion of the fibrin glue blocks in the saline solution, high bacteriophage numbers were released (Fig. 5). Beginning at the second hour, significantly more (P = 0.0079) phages were released from fibrin glue blocks with embedded bacteriophages than the control blocks soaked in a bacteriophage solution. All fibrin glue samples showed mechanical stability during the 7 day incubation period. Visual deterioration of the all samples began on day 8 and all blocks were completely dissolved by day 11. Complete dissolution corresponded to release of all remaining bacteriophages from the fibrin samples. At this time point, the titer of the released phages from embedded bacteriophage samples was the highest. At the last time point, phage titers from the bacteriophage-soaked samples also increased, but was not more than its initial level (1 hour).

### Antibacterial efficacy of released bacteriophages

**Figure 6:**
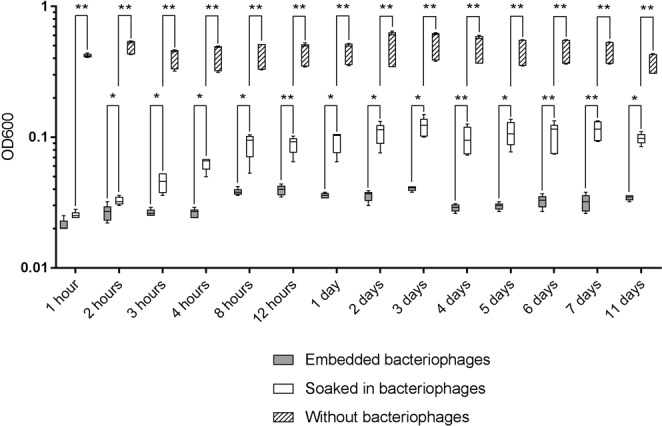
OD600 measurements after co-incubation of fibrin glue-released PA5 phages with *P*. *aeruginosa* PA01. Comparison of fibrin glue blocks with embedded bacteriophages to control samples where fibrin glue blocks were soaked in a solution of PA5 phage. OD600 values are presented as the mean (n = 5) ± standard deviation. *p < 0.05; **p < 0.01; ***p < 0.001.

Using the spot-test method, fibrin glue blocks with embedded bacteriophages and blocks soaked in bacteriophages produced clear zones of lysis at all time points indicating effective bacterial killing. Measurement of the OD600 of *P*. *aeruginosa* PA01 culture (Appleman’s method) showed significantly higher antibacterial effect of the phages from the embedded bacteriophages beginning at hour 2 (P = 0.0459) (Fig. 6).

**Figure 7:**
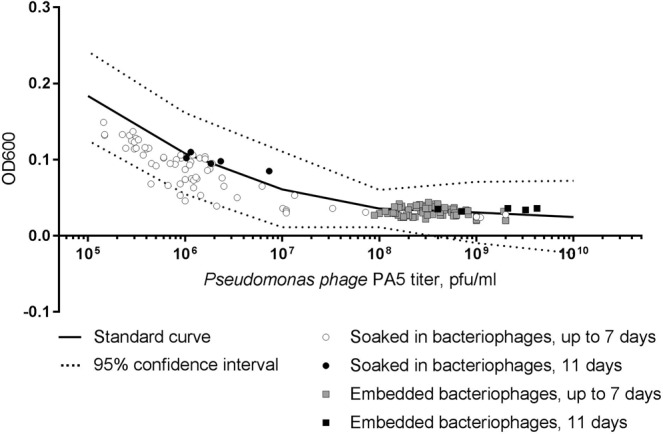
Relationship between PA5 phage titer and the *P*. *aeruginosa* PA01 culture OD600. The black line represents a standard curve of a serial dilution of the stock PA5 phage. The dot plot corresponds to the released phage titer and OD600 measurement.

In the process of determining phage titers, bacteriophages, and consequentially fibrin, are serially diluted. The relationship between released phage titer and antibacterial efficacy was investigated in order to confirm that the infectivity of bacteriophages was not affected by high concentrations of fibrin. This may be important especially in the final days of fibrin glue deterioration due to presence of high concentrations of dissolved fibrin in the samples. On the other hand residual fibrin glue may potentially inhibit bacterial growth^20^. Viability of released PA5 bacteriophages from both embedded and soaked fibrin glue blocks with different titers showed that *P*. *aeruginosa* PA01 culture OD600 values of all samples fell within the 95% confidential interval of the standard serial dilution curve (Fig. 7). Together, these data confirm stability of phages released from fibrin glue with titer-dependent lytic activity comparable to native bacteriophage preparations.

### Diffusion of bacteriophages within fibrin glue

**Figure 8:**
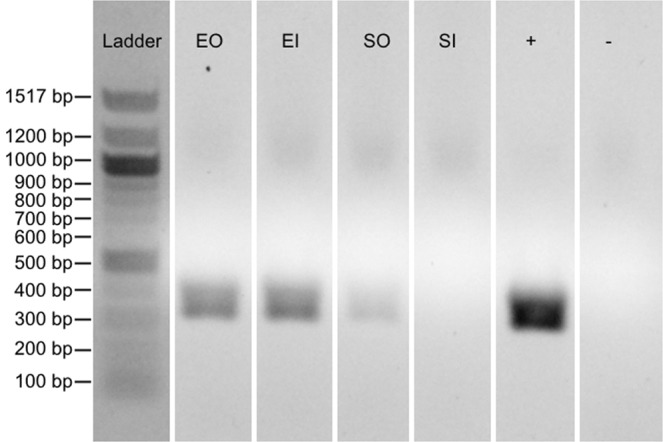
Presence of the PA5 phage in fresh fibrin glue scaffolds. Confirmation of the phage distribution along fibrin glue blocks was performed by puncture of the blocks followed by a phage-specific PCR of the sample. Ladder, DNA molecular weight marker; EO, embedded bacteriophages sample outside; EI, embedded sample inside; SO, sample soaked in bacteriophages outside; SI, sample soaked in bacteriophages outside; +, positive control; −, negative control. Full-length gel is presented in Supplemental Fig. S2.

We found that bacteriophages were well distributed within fibrin glue scaffolds when embedded into the fibrin glue. On the other hand, when soaked in a phage solution, bacteriophages were initially only detected within the outer layer of the scaffold (Fig. 8). However, after one month storage at 4 °C, phages were detected equally within the inner and outer layers of all fibrin glue scaffolds (Supplemental Fig. S2). Based on both PCR and phage release results we conclude there is diffusion of undamaged bacteriophage virions from superficial to deeper parts of the fibrin glue scaffolds in the case of the soaked control samples (Supplemental Fig. S3).

## Discussion

Despite asepsis and systemic prophylactic administration of antibiotics, wound and surgical implant infections are relatively common^5,21–23^. Intravenous antibiotics are often unable to reach sufficient topical tissue concentration and even when they do, bacteria are often protected within biofilms. Fibrin glue is clinically useful in a wide variety of surgical specialties as a hemostat, sealant, and adhesive. Fibrin glue has also been explored as a means of drug delivery. Drugs incorporated into fibrin glue enable sustained site-directed release from the self-dissolving matrices. Several recent studies have found that incorporation of antibiotics into fibrin glue facilitates effective, site-directed, sustained antimicrobial activity^15–17^. However, considering the global rise in antibiotic resistance and the increasing awareness of the importance of human microbiota, there has been increased interest in bacteriophage therapy. Bacteriophages target specific bacteria, leaving the host’s own microbiota undisturbed^10,24^. They are self-amplifying “auto dosing” antimicrobials in that they replicate themselves as long as there are host bacteria to infect and they are particularly effective at penetrating and disrupting biofilms^11^.

Unlike other drugs incorporated into fibrin glue, bacteriophages carry genetic material that needs to be protected to preserve activity. We have now tested the stability and viability of bacteriophages incorporated in fibrin glue and quantified antibacterial activity of released bacteriophages. The PA5 bacteriophage showed good biocompatibility (survival) within fibrin glue. Therefore, bacteriophage-loaded fibrin glue appears to a promising antibacterial-delivery system that could be used to treat antibiotic-resistant bacteria.

A number of different carriers for bacteriophages have been tested in other studies including liposomes, transfersomes, and different polymeric scaffolds^25–29^. Many of these carriers seem to be promising for application of fixed phage formulations. However, there is a current need phage therapy for healthcare-associated infections for the application of customizable, personalized (magistral or compounding tailor made) preparations^7,10^. Fibrin glue has the potential to be an ideal carrier for these purposes because of its suitability for on-demand preparations and its availability in hospitals. Application of bacteriophages in combination with fibrin glue might also be promising from an immunological point of view. Bacteriophages stimulate phagocytosis activity of neutrophils and promote complete phagocytosis^30^. Moreover, fibrin glue is postulated to improve local cellular immunity by stimulating phagocyte motility^20^. In this way a synergistic effect may be achieved^31^. On the other hand, there may be challenged to face when considering long-term use of bacteriophages embedded in fibrin glue. For example, bovine aprotinin, which is used in TISSEEL, is immunogenic in humans and anti-aprotinin IgG antibodies are prevalent in up to 80% of patients 3.5 months after exposure to both local and intravenous aprotinin during cardiac surgery^32^. In addition, bacteriophages are in general immunogenic and anti-phage antibodies can neutralize bacteriophages resulting in reduced efficacy of phage therapy over prolonged periods^33–36^. Fusion of phage tails with fibrin fibers could potentially protect phages from neutralizing antibodies, however, further studies are required to examine this theory.

We used relatively high bacteriophage titers based on our preliminary experiments and to ensure interaction between the PA5 phage and *P*. *aeruginosa* PA01. Our EM images allowed us to visualize the incorporation and distribution of bacteriophages in fibrin glue scaffolds. SEM with contrasting metals or cryo-EM could be useful in understanding this interaction even further. Future EM experiments will also track dissolution of bacteriophage-fibrin glue matrices over time.

Fibrin glue can be extruded from a syringe or sprayed to cover surfaces (eg. tissue or implanted medical devices). We foresee no difficulties in using bacteriophage-loaded fibrin glue in spray applications, however, it must be tested. From our results comparing embedded bacteriophages to soaking pre-formed fibrin glue matrices in bacteriophage solutions, we would predict that incorporating bacteriophages into the fibrin glue spray would have advantages over soaking fibrin glue-coated devices in bacteriophage solutions. We observed a more sustained release of viable bacteriophage when they were embedded upon glue synthesis.

Future work will test whether the combination of bacteriophages and antibiotics within fibrin glue have an additive antibacterial effect. In addition, various clinically-relevant lytic phages of different sizes and morphologies also needed to be evaluated. Overall, our results show that fibrin glue can be used for sustained delivery of bacteriophages and shows promise to be an effective prophylaxis for surgical and implant infections. Therefore, the combination of bacteriophages in fibrin glue could be a new tool in the surgeon’s toolbox.

## Methods

### Bacteriophage and bacterial strains

The well-characterized lytic *Pseudomonas* phage PA5 (family *Myoviridae*; genus *Pbunavirus*; GenBank accession no.: KY000082.1) was used as a model to test the suitability of fibrin glue for phages (Fig. 3)^37^. The PA5 phage has a virion length of ≈143 nm and a head diameter of 40–52 nm. *Pseudomonas aeruginosa* strain PA01 (ATCC 27853) was used as the host bacteria to propagate the PA5 phage and for assessing the phage’s antibacterial activity. Trypticase soy broth (TSB) and agar (TSA) were used for maintenance of the PA01 stocks.

Preparation of bacteriophage stocks used the solid agar media method with slight modifications^38^. Briefly, a fresh overnight broth culture of the *P*. *aeruginosa* PA01 was inoculated on top of TSA inside Roux flasks. After a 2.5 h incubation at 37 °C, excess liquid was discarded from the flasks, the PA5 phage was inoculated on top of the preformed growing lawn, and the flasks were incubated for 15 h at 37 °C. Amplified bacteriophages were washed from the TSA surface with 5–10 ml of SM buffer and bacteria were removed by filtration through a 0.22 µm polyethersulfone syringe filter (Sarstedt AG, Germany).

Phage stocks were concentrated and purified using Vivaspin 20 ultrafiltration units with a molecular weight cutoff of 1000 kDa (Sartorius AG, Germany). After the final concentration step, purified bacteriophages were resuspended in sterile 0.9% NaCl.

### Incorporation of bacteriophages into fibrin glue

We tested the ready-to-use surgical fibrin sealant TISSEEL (Baxter, USA) which consists of two syringes loaded with equal volumes of fibrinogen and thrombin. These components meet together in a common outlet at the time of the fibrin glue application. As a result, thrombin enzymatically converts fibrinogen to fibrin. We substituted half of the thrombin volume with either 0.9% NaCl or 1.13 × 10^11^ bacteriophages suspended in 0.9% NaCl.

Fibrin glue scaffolds were created by filling a sterile silicon tube (inner diameter, 6 mm) and were allowed to polymerize for 5 min at room temperature. The control and bacteriophage-containing fibrin glue scaffolds were cut into 10 mm length blocks (Fig. 2). Each 6 × 10 mm block contained 1.88 × 10^10^ pfu.

### Scanning electron microscopy

Fibrin glue blocks (±bacteriophages) were fixed in 2.5% glutaraldehyde and sent for SEM. SEM images were captured with an FEI Versa scanning electron microscope (Thermo Fisher Scientific, USA) using a low accelerating voltage of 1 kV and a beam current 7.4–27 pA or in a low vacuum mode with a SEM chamber pressure of 100 Pa to avoid of charging effects. No special sample preparation procedures like a conductive coating deposition were performed.

### Assessment of bacteriophage release and antimicrobial ability

Control fibrin glue blocks were incubated for 30 minutes at 37 °C with 1.13 × 10^11^ pfu in 0.9% NaCl. For assessment of the bacteriophage release, fibrin glue blocks were incubated in 3 ml of 0.9% NaCl at 37 °C with permanent rotation at 200 rpm. Supernatants were harvested and fresh 0.9% NaCl was given at the following time points: 1, 2, 3, 4, 8, 12, 24 hours, and 2, 3, 4, 5, 6, 7 days. After the last time point, samples were incubated until complete dissolution of the fibrin scaffold.

The conventional double agar overlay method was used to measure titer of the bacteriophages released into the supernatant^24,39^. Qualitative assessment of the released PA5 bacteriophage was performed using the conventional spot-test method^40,41^. We used 10 µl of 0.9% NaCl with released phages from each sample to test their ability to lyse the host-strain.

The Appelmans method was used to test antibacterial activity of the released PA5 phage^42^. Briefly, 1 ml of the 0.9% NaCl containing released bacteriophages was mixed with 1 ml of 2 × LB broth and added to a 100 ml *P*. *aeruginosa* PA01 culture containing 1 × 10^7^ CFU. The mixtures were incubated for 4 hours at 37 °C with rotation at 200 rpm. The OD600 was measured with a BioPhotometer D30 device (Eppendorf, Germany).

### Polymerase chain reaction

Fibrin glue scaffolds soaked in bacteriophages or containing embedded bacteriophages were either frozen at −70 °C directly or after one month storage at 4 °C. After freezing, scaffolds were cleaved in half to prevent phage cross-contamination between the inner and outer layers. The inner of outer surface of the scaffolds were punctured (depth 2 mm) with sterile syringe needles (diameter 0.7 mm). Needles were then washed with sterile 0.9% NaCl and the solution was treated with DNAse I (New England Biolabs, USA) to remove all phage-free DNA. Nucleic acids were extracted using the QIAamp MinElute Virus Spin Kit (QIAGEN, Germany). PA5-specific primers were used (PA5_fw2, 5-ATCAGCAAGACCCATTCGC-3; PA5_rv2, 5-AGCCAACGGTATCGATGCC-3). Each PCR reaction (25 µl) contained 5 µl of 5 × Q5 Reaction Buffer, 0.5 units of Q5 High-Fidelity DNA polymerase (New England Biolabs, USA), 200 nM dNTPs, 0.5 µM of each primer, and 2 µl of the phage DNA. PCR conditions were as follows: 4 min at 98 °C followed by 35 cycles at 98 °C for 10 s, 66 °C for 30 s, and 72 °C for 40 s. PCR was performed with T100 thermocycler (Bio-Rad, USA). PCR products were analyzed on a 2% agarose gel.

### Statistical analysis

Data were analyzed using GraphPad Prism 7. The primary hypothesis was equal efficacy (OD600 extinction) of control and experimental samples; the secondary hypothesis was that the amount of phage released from both groups were equal. Pairwise comparisons of control and experimental samples at each time point were done with Mann-Whitney-U-tests because the distribution of both released phages and OD600 absorption, were not consistently normally distributed. Hierarchically ordered hypotheses (according to descending experiment duration) permitted to keep the significance level at 5% up to the first nonsignificant comparison. For further group comparisons we also used Mann-Whitney-U-tests.

## Supplementary information

Supplementary Materials
