# Supplementary material for: Fibrin glue as a local drug-delivery system for bacteriophage PA5
Source: Sci Rep. 2019 Feb 14;9:2091. doi: 10.1038/s41598-018-38318-4 (PMC6376040; doi:10.1038/s41598-018-38318-4)
Supplement: Supplementary file 1 — Supplementary Materials [file 41598_2018_38318_MOESM1_ESM.pdf]

Supplementary Materials

Fibrin glue as a local drug-delivery system for bacteriophage PA5

Evgenii Rubalskii, Stefan Ruemke, Christina Salmoukas, Andrey Aleshkin, Svetlana Bochkareva, Evgeny Modin, Bakr Mashaqi, Erin C. Boyle, Dietmar Boethig, Maxim Rubalsky, Eldar Zulkarneev, Christian Kuehn, Axel Haverich

*Corresponding author, e-mail: rubalskii.evgenii@mh-hannover.de, fax:+495115325404

Bacteriophage virions were not observed on the surface of the experimental blocks (Supplemental Fig. S1).

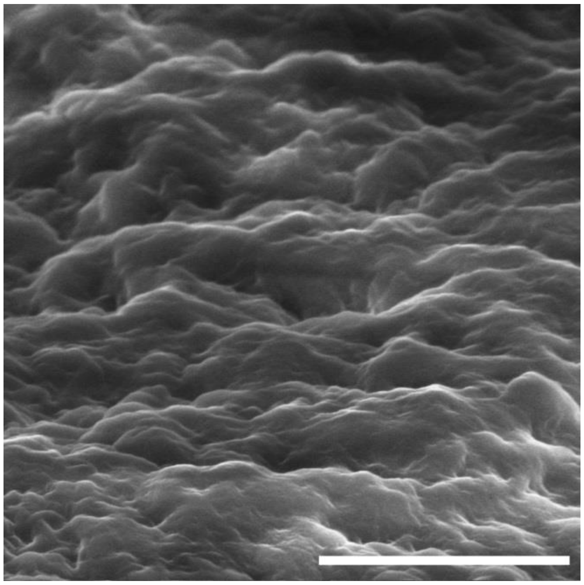

Supplemental Figure S1. Electron microscopy of the outer surface of the fibrin glue block. Scale bar, 2 µm.

After one month storage at 4°C, phages were detected within both the inner and outer layers of all fibrin glue scaffolds (Supplemental Fig. S2).

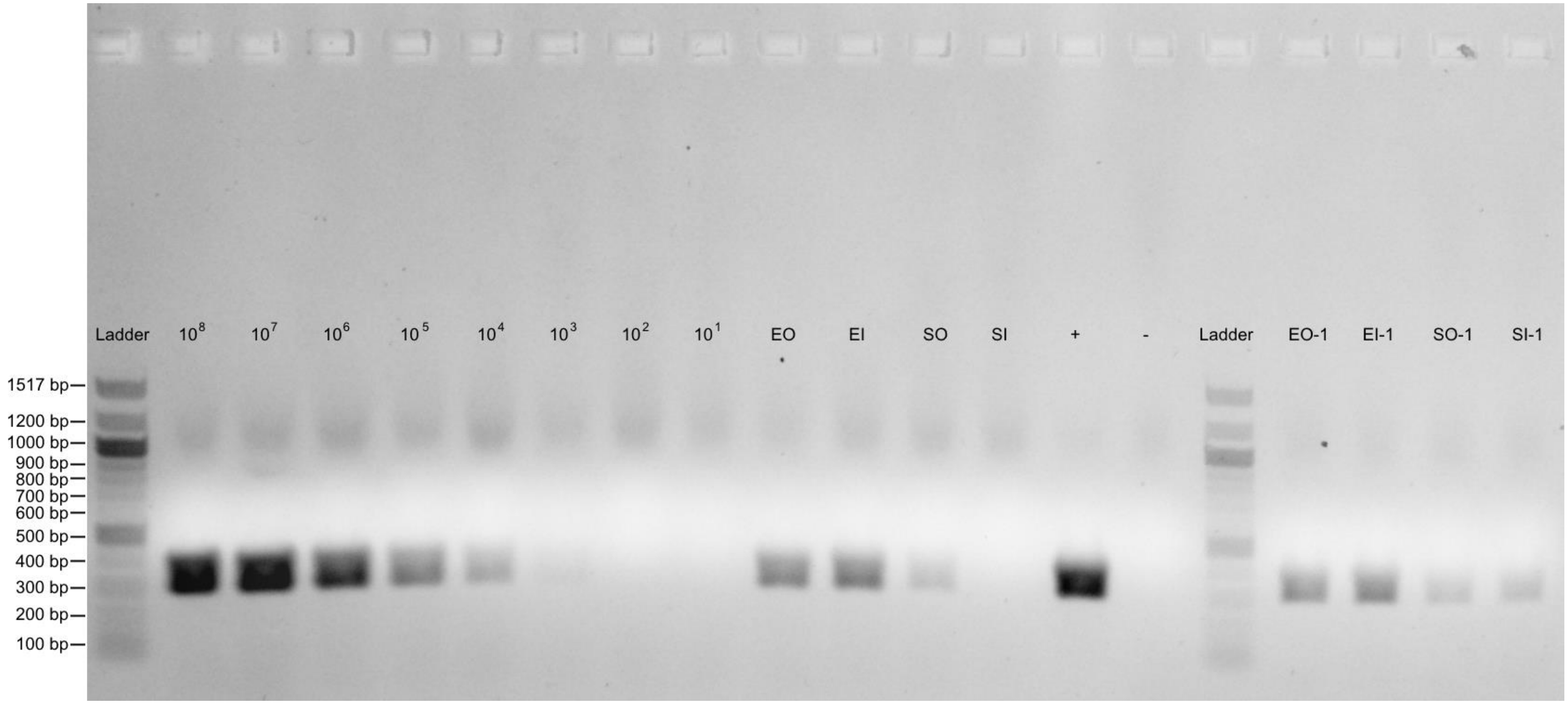

Supplemental Figure S2. Presence of the PA5 phage in fibrin glue scaffolds before and after one month storage. Ladder, DNA molecular weight markers. Lanes 2 to 9 correspond to standards containing 10^8 to 10^1 copies of PA5 DNA per ml respectively. EO, embedded bacteriophages sample outside before storage; EI, embedded sample inside before storage; SO, sample soaked in bacteriophages outside before storage; SI, sample soaked in bacteriophages outside before storage; +, positive control; -, negative control; EO-1, embedded bacteriophages sample outside after storage; EI-1, embedded sample inside after storage; SO-1, sample soaked in bacteriophages outside after storage; SI-1, sample soaked in bacteriophages outside after storage.

Based on both PCR and phage release profiles, results we observed diffusion of undamaged bacteriophage virions from the superficial to the deeper parts of the fibrin glue scaffolds (Supplemental Fig. S3). According to these data it is possible to assume movement of the phages inside of the fibrin glue and outside of it in the direction opposite to their concentration gradient vector.

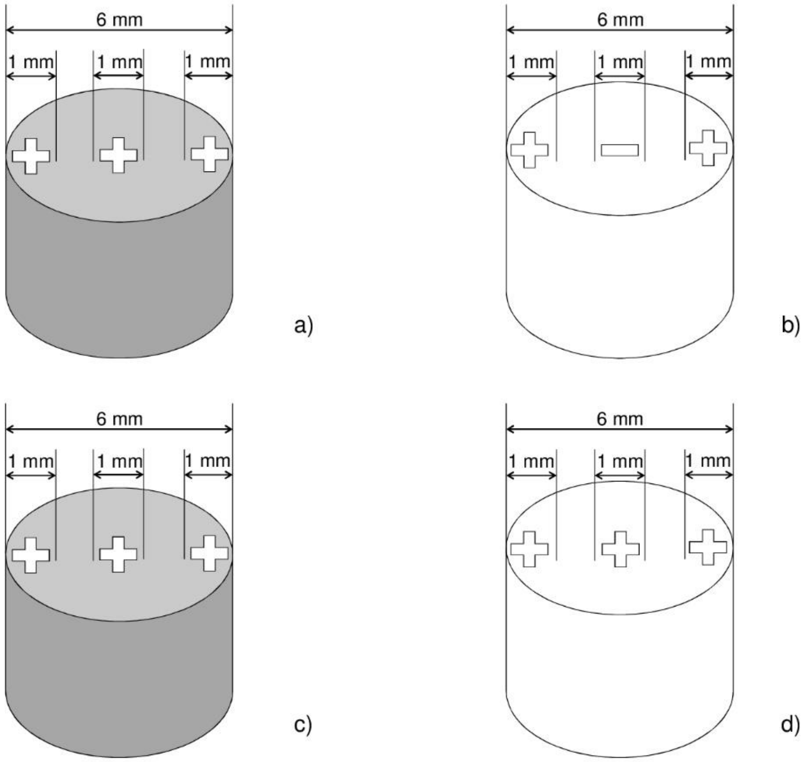

Supplemental Figure S3. Schematic of the presence of PA5 phage in different sites within the fibrin glue scaffolds. a) Fresh fibrin glue embedded with bacteriophages; b) Fresh fibrin glue soaked in bacteriophages; c) Fibrin glue with embedded bacteriophages after one month of storage; d) Fibrin glue soaked in bacteriophages after one month of storage. “+”, phage PA5 detected; “-”, phage PA5 not detected.

Summation of the absolute number of released phages showed significantly higher phage load of the fibrin glue blocks embedded with bacteriophages after 12 hours of the incubation (P=0.0317) (Supplemental Fig. S4).

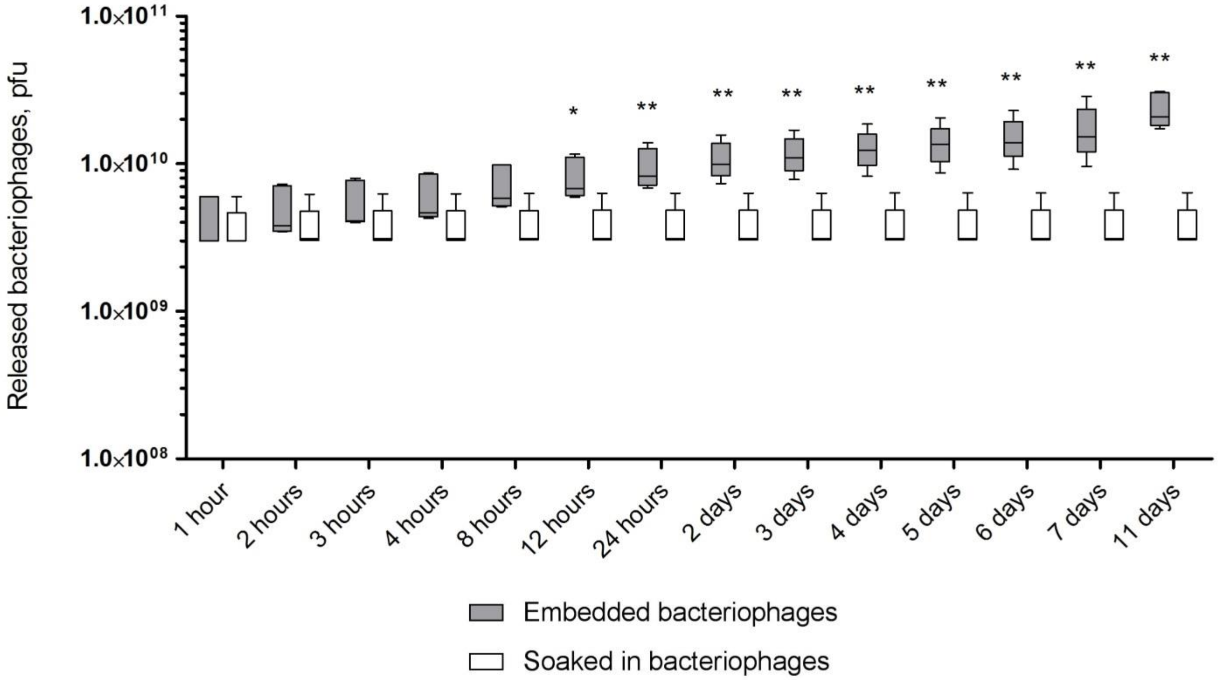

Supplemental Figure S4. Cumulative *P. aeruginosa* PA5 phage release from fibrin glue blocks. Comparison of fibrin glue blocks with embedded bacteriophages to control samples where fibrin glue blocks were soaked in a solution of PA5 phage. Numbers of released phages are presented as the mean (n=5) ± the standard deviation. * $p<0.05$; ** $p<0.01$; *** $p<0.001$.
